# A NOVEL DNA METHYLATION-BASED SURROGATE BIOMARKER FOR LATENT SYSTEMIC INFLAMMATION

**DOI:** 10.1093/geroni/igae098.4292

**Published:** 2024-12-31

**Authors:** Helen Meier, Eric Klopack, Mateo Farina, Belinda Hernández, Colter Mitchell, Jessica Faul, Cathal McCrory, Eileen Crimmins

**Affiliations:** Institute for Social Research, Institute for Social Research, Michigan, United States; University of Southern California, University of Southern California, California, United States; University of Texas at Austin, Austin, Texas, United States; Trinity College Dublin, Dublin, Dublin, Ireland; University of Michigan, Ann Arbor, Michigan, United States; University of Michigan, Ann Arbor, Michigan, United States; Trinity College Dublin, Dublin, Dublin, Ireland; University of Southern California, Los Angeles, California, United States

## Abstract

Chronic low-grade systemic inflammation is a risk factor for chronic diseases and mortality and is an important biomarker in health research. DNA methylation (DNAm) surrogate biomarkers are valuable exposure, risk factor and health outcome predictors in population studies. We generated a DNAm surrogate biomarker for chronic, systemic inflammation from a previously identified systemic inflammation latent variable derived from seven inflammatory markers and evaluated its performance relative to measured inflammatory biomarkers in predicting several age-associated outcomes of interest, including mortality, activities of daily living and multimorbidity in the Health and Retirement Study. The DNAm surrogate, Inflammation Latent Variable Methylation Surrogate (InfLaMeS), strongly correlated with individual inflammation markers and performed similarly to the latent systemic inflammation when predicting multimorbidity, disability, and 4-year mortality. These results suggest that InfLaMeS provides a robust alternative to blood-chemistry measures of inflammation that can be used for further scientific insight into understanding the role of inflammation in aging and health.

